# Web-Based, Human-Guided, or Computer-Guided Transdiagnostic Cognitive Behavioral Therapy in University Students With Anxiety and Depression: Randomized Controlled Trial

**DOI:** 10.2196/50503

**Published:** 2024-06-19

**Authors:** Jurrijn Koelen, Anke Klein, Nine Wolters, Eline Bol, Lisa De Koning, Samantha Roetink, Jorien Van Blom, Bruno Boutin, Jessica Schaaf, Raoul Grasman, Claudia Maria Van der Heijde, Elske Salemink, Heleen Riper, Eirini Karyotaki, Pim Cuijpers, Silvia Schneider, Ronald Rapee, Peter Vonk, Reinout Wiers

**Affiliations:** 1 Department of Developmental Psychology University of Amsterdam Amsterdam Netherlands; 2 Technical Support Psychology Department University of Amsterdam Amsterdam Netherlands; 3 Cognitive Neuroscience Department Donders Institute for Brain, Cognition and Behaviour Radboud University Nijmegen Netherlands; 4 Department of Psychological Methods University of Amsterdam Amsterdam Netherlands; 5 Department of Research, Development and Prevention Student Health Service University of Amsterdam Amsterdam Netherlands; 6 Experimental Psychopathology and Clinical Psychology Lab Department of Clinical Psychology Utrecht University Utrecht Netherlands; 7 Amsterdam Public Health Research Institute Department of Clinical Neuro- and Developmental Psychology Vrije Universiteit Amsterdam Netherlands; 8 Mental Health Research and Treatment Center Department of Clinical Child and Adolescent Psychology Ruhr-Universität Bochum Bochum Germany; 9 Centre for Emotional Health Department of Psychology Macquarie University Sydney Australia

**Keywords:** internet-based cognitive behavioral intervention, iCBT, university students, transdiagnostic, human guidance, technological guidance

## Abstract

**Background:**

Internet-based cognitive behavioral interventions (iCBTs) are efficacious treatments for depression and anxiety. However, it is unknown whether adding human guidance is feasible and beneficial within a large educational setting.

**Objective:**

This study aims to potentially demonstrate the superiority of 2 variants of a transdiagnostic iCBT program (human-guided and computer-guided iCBT) over care as usual (CAU) in a large sample of university students and the superiority of human-guided iCBT over computer-guided iCBT.

**Methods:**

A total of 801 students with elevated levels of anxiety, depression, or both from a large university in the Netherlands were recruited as participants and randomized to 1 of 3 conditions: human-guided iCBT, computer-guided iCBT, and CAU. The primary outcome measures were depression (Patient Health Questionnaire) and anxiety (Generalized Anxiety Disorder scale). Secondary outcomes included substance use–related problems (Alcohol Use Disorder Identification Test and Drug Abuse Screening Test—10 items). Linear mixed models were used to estimate the effects of time, treatment group, and their interactions (slopes). The primary research question was whether the 3 conditions differed in improvement over 3 time points (baseline, midtreatment, and after treatment) in terms of depression and anxiety symptoms. Results were analyzed according to the intention-to-treat principle using multiple imputation. Patients were followed exploratively from baseline to 6 and 12 months.

**Results:**

In both short-term and long-term analyses, the slopes for the 3 conditions did not differ significantly in terms of depression and anxiety, although both web-based interventions were marginally more efficacious than CAU over 6 months (*P* values between .02 and .03). All groups showed significant improvement over time (*P*<.001). For the secondary outcomes, only significant improvements over time (across and not between groups) were found for drug use (*P*<.001). Significant differences were found in terms of adherence, indicating that participants in the human-guided condition did more sessions than those in the computer-guided condition (*P*=.002).

**Conclusions:**

The transdiagnostic iCBT program offers a practical, feasible, and efficacious alternative to usual care to tackle mental health problems in a large university setting. There is no indication that human guidance should be preferred over technological guidance. The potential preference of human support also depends on the scale of implementation and cost-effectiveness, which need to be addressed in future trials.

**Trial Registration:**

International Clinical Trials Registry Platform NL7328/NTR7544; https://trialsearch.who.int/Trial2.aspx?TrialID=NL-OMON26795

## Introduction

### Background

University students face various challenges, both academically and personally [1]. During the phase of emerging adulthood (18-25 years of age), difficulties may arise in facing these challenges [2]. Recent large-scale studies among undergraduate students have shown that between 20% and 30% of students experience a mental disorder, most notably depression and anxiety disorders [3-5]. In addition, student life has been associated with excessive alcohol consumption, with some studies indicating that 20% of students are hazardous drinkers [6]. Symptoms of common mental disorder impact the ability to face key challenges of emerging adulthood, such as identity formation and building new, intimate relationships [7,8]. Mental distress may also contribute to students performing poorly academically or even dropping out. This may give rise to concerns about career prospects and finding a place in society [7-9]. These dynamics may lead to a vicious cycle of increasing feelings of failure, which may further exacerbate occurring symptoms. Thus, timely intervention in this group is needed [10,11].

Despite the availability of local services at or around the university, most students do not receive the help they need, and some do not even seek help in the first place [12]. Auerbach et al [4], based on surveys in 21 countries, found that less than 20% of students with a recent or current mental disorder received *minimally adequate treatment* for this disorder, and this rate is even lower in lower middle–income to low-income countries. Even with severe symptoms of a common mental disorder, the 12-month treatment rates did not exceed 45.1%, meaning that half of all students with longer-lasting severe symptoms do not receive help for their problems [12]. Impediments to find help include both factual (eg, lack of skilled therapists, waitlists, lack of time, and financial hurdles) and perceived barriers (eg, skepticism on treatment effectiveness, lack of perceived urgency, and fear of stigmatization) [13-17]. High levels of unmet treatment needs emphasize the urgency to explore alternative possibilities for intervention and potentially a reallocation of resources for this vulnerable group [12].

To address some of these barriers, innovative, scalable, and low-threshold interventions are required. Web-based treatment may meet some of these requirements and has been shown effective for this target group, particularly for depression and anxiety [18-20]. Another potential advantage of web-based interventions is that they require less human investment in terms of time compared with face-to-face clinical care and thus may reduce costs, although this should be quantified in future studies explicitly aimed at assessing cost-effectiveness (not the objective of this study). Moreover, young adults may prefer easily accessible and relatively anonymous and self-directed interventions with limited therapeutic contact [14,17]. Meta-analytic findings have indicated that older people are more likely to respond better to web-based treatments for depression; thus, methods to increase efficacy for young adults need to be found [21].

Transdiagnostic web-based approaches may broaden the scope and impact of interventions. This is particularly relevant given the high comorbidity of symptoms among students [5]. There is a growing consensus that depression and anxiety, although often classified as distinct disorders, share common etiological and perpetuating factors, such as proneness to internalization and high levels of negative affect [22,23]. Targeting anxiety and depression in one treatment program may appeal to a large variety of students. However, at the same time, studies using web-based transdiagnostic interventions among students have reached inconsistent findings, although these studies had limited scope and compared treatment to a waitlist control group only [24,25]. In general, the potential of transdiagnostic interventions for large-scale implementation should not be underestimated, and they could have secondary effects on related problems, such as substance use issues [26].

Evidence is accruing that *guided* (cognitive behavioral therapy [CBT]) interventions should be preferred over *unguided* or self-guided interventions with only limited (ie, technological) support, given their greater efficacy and positive impact on adherence [27,28]. Karyotaki et al [27], for example, found some advantageous effects of guided versus unguided internet-based CBT (iCBT) for depression, but these differences disappeared at the 6-month and 12-month follow-up. This has important practical implications, as offering human support exerts greater pressure on extant resources and could thus limit implementation on a large scale (ie, in a university setting). Guided and unguided web-based interventions have rarely been compared head-to-head among students with mental health issues [28]. In this study, we use a slightly different terminology for the guided and unguided iCBT conditions, as we think these terms are unfortunate for several reasons (for a discussion, see the study by Koelen et al [28]).

### Aims of This Study

In this study, we compared 3 conditions simultaneously in a large sample of both undergraduate and graduate (including doctoral) university students (henceforth, students): a human-guided iCBT transdiagnostic program, a computer-guided iCBT transdiagnostic program, and care as usual (CAU). This study built upon a previous study [10] comparing a human-guided (transdiagnostic) iCBT against CAU in a sample of students (n=100). This previous study [10] yielded no significant differences (*P*>.05) between conditions on any of the examined outcomes, at any of the time points (including the 6-month and 12-month follow-up), which may in part be attributable to limited power to detect small effects. In addition, this previous study [10] included only students with mild to moderate anxiety and depression symptoms. As other iCBT trials indicate that interventions are more efficacious when offered to individuals with higher levels of anxiety and depression symptoms [25,27], we also included students with elevated levels of anxiety and depression beyond mild symptoms in this study.

The aims of this study were two-fold: (1) to examine the efficacy of a transdiagnostic iCBT program in a large sample of students with mild, moderate, or severe levels of anxiety, depression, or both, when compared with CAU, and (2) to examine whether the addition of human guidance resulted in greater efficacy when compared with computer guidance. It was expected that (1) individuals who followed either human-guided or computer-guided iCBT would improve more than those in the CAU condition on the primary outcomes of depression and anxiety after treatment and (2) individuals who followed human-guided iCBT would improve more on the primary measures compared with computer-guided iCBT. Exploratory analyses were performed up until 6 and 12 months after baseline and for the secondary outcomes. Higher adherence rates were expected for the human-guided compared with the computer-guided condition [28].

## Methods

### Design

This study was a 3-arm, randomized controlled superiority trial conducted at the University of Amsterdam (UvA) in the Netherlands. A web-based, personalized, and transdiagnostic intervention (iCare Prevent) [29] with human guidance was compared with the same intervention with computer guidance. Both web-based interventions were also compared with CAU. CAU in this context refers to the standard mental health care that is accessible in the university setting, including help provided by the (student) general practitioners, student psychologists, and study advisers, as well as the secondary services in the broader community (psychologists and psychiatrists). Participants were followed up to 12 months after randomization. Measurements were administered at baseline (t1); midtreatment (5 weeks after randomization; t2); after treatment (8 weeks after randomization; t3); 6-month follow-up (t4); and 12-month follow-up (t5).

### Ethical Considerations

This study was approved by the medical ethical committee of the *Centrale Commissie Mensgebonden Onderzoek* (METC number 2018_085, NL64929.018.18).

### Participants

Participants were young adults who were enrolled as bachelor’s, master’s, or PhD students at the UvA. Before inclusion in the randomized controlled trial, students participated in an e-survey to screen for elevation of symptoms of anxiety or depression (mild, moderate, or severe) [30]. Students were recruited between February 2019 and March 2022 and were included in the randomized controlled trial based on the following inclusion criteria: (1) aged ≥16 years and (2) mild, moderate, or severe symptoms of depression (as defined by a score within the range of 15 to 60 points on the Center for Epidemiological Studies Depression Scale) [31], anxiety (as defined by scoring above the cut-off score of 4 on the Generalized Anxiety Disorder scale–7 items [GAD-7]) [32] on the e-survey, or both.

Participants were excluded when they fulfilled one or more of the following criteria: (1) comorbid bipolar disorder or psychotic disorder according to the Mini International Neuropsychiatric Interview (MINI) [33]; (2) active high suicide risk according to the MINI; (3) currently receiving psychological treatment for depression or anxiety; (4) having a slow or no internet connection; and (5) no provision of providing written informed consent before participation.

### Procedures of Screening and Intervention Phases

Students enrolled at the UvA received an invitation via email from the secured, central research-dedicated platform (LOTUS). LOTUS contained the students’ email addresses, from which links for the e-survey platform Qualtrics and other messages were sent, depending on the phase of the study and the forms completed. The invitation email contained general information about the study and a unique link. Participants who clicked on the invitation link were referred to the e-survey platform. Here, they could find an information letter and were asked to provide informed consent and complete the survey. Invitations were sent in separate cohorts to control the participant flow throughout the study’s distinct phases. Following the original invitation, 2 reminder emails were sent (1 and 2 weeks after the first invitation). In addition, study advisers and study counselors of the UvA were informed about the study. They were asked to refer students interested in participating in the study to the research team. The study spanned 4 academic years (2018/2019 to 2021/2022), and participants were asked at the beginning of the screening if they consented to being invited again later that academic year. Participants could opt out of the study at any time, and they could also indicate that they did not want to receive further emails. During the COVID-19 pandemic (April 2020 until the end of the study), the screening e-survey contained several additional questions about their activities and coping style (for details, see the study by Koelen et al [34]).

Participants who scored above the cut-off on either anxiety symptoms, depressive symptoms, or both received an email with a web-based information brochure and informed consent document. Participants could also choose to receive the information brochure and informed consent document through the post. Likewise, informed consent could be signed on the web, returned by post, or handed in at several *collection points* at the university campus. Next, they were telephoned by the administrators of the project to book an appointment to conduct the MINI diagnostic interview (see the *Measures* section). When eligibility was confirmed, participants were immediately randomized to one of the 3 conditions. The participants who were randomized to either intervention were instructed to create an account on the intervention platform (Minddistrict).

Participants were reimbursed for completing the 6-month and 12-month follow-up assessments. In the initial phases of the trial, participants were paid €10 (US $10.8) when they completed both assessments. To increase adherence, students were paid €5 (US $5.4) for each of the assessments separately in the final stages of the trial. A raffle to win a tablet or e-reader was held among every hundred participants that completed both the 6-month and 12-month assessments.

### Randomization, Blinding, and Treatment Allocation

Participants who were eligible to participate in the study were randomly assigned to either the human-guided intervention, the computer-guided intervention, or CAU (1:1:1 allocation ratio) group directly following the baseline measurement, which was done automatically through an algorithm built into the LOTUS platform. Randomization was based on computer-generated random numbers. Participants were stratified by gender and anxiety and depressive symptoms to guarantee an even distribution of male and female participants and symptom severity across conditions. Allocation was concealed from psychologists involved in this study; 2 of the psychologists who provided guidance were also administrators with access to the backend of the software. Owing to the nature of the intervention, participants and psychologists could not be blinded to the assigned treatment condition. Participants were informed about the 3 conditions and whether they were assigned to either an intervention condition or CAU. It was not specified to participants to which intervention (human-guided or computer-guided) they were assigned. Psychologists were aware of the participants’ intervention condition because they provided participants with personalized feedback in the human-guided condition. The assessments were all conducted on the web and were not accessible for the psychologists (ie, blinded), except for the presession questions that were available through the intervention platform.

### Interventions

The web-based transdiagnostic intervention that was used in this study, *iCare Prevent*, was originally developed by Weisel et al [35] for the German-speaking general population and translated and adapted by Bolinski et al [29] for a Dutch undergraduate student population into Dutch and English (see Textbox 1 for modules of iCare Prevent). For this study, we created a second English version of the intervention for PhD students that included a small adaptation of the examples to match their situation. The intervention is based on principles from CBT for anxiety and depression and includes web-based exercises and homework assignments. The intervention comprised 7 regular sessions (45-60 min/session) and 1 booster session (4 weeks after the completion of the last session). From the second session onward, participants were able to follow 8 additional optional modules based on their personal needs, including sleep, perfectionism, alcohol use, rumination, self-worth, acceptance, appreciation and gratitude, and rest and relaxation. In sessions 5 and 6, participants could decide to either engage in content directed at changing negative cognitions or at exposure to fear situations. They could decide to choose 1 additional module per session, and they were free to repeat this module or choose other modules in later sessions. It was advised to do at least 1 and no more than 2 treatment sessions per week. For a full description of the intervention, see the study by Karyotaki et al [10].

**Overview of content of the iCare Prevent training.**

**Sessions**

1. Behavioral activation: reducing incongruence
2. Behavioral activation: overcoming difficulties and pleasant activity scheduling
3. Psychoeducation
4. Cognitive restructuring
5. Problem solving I or exposure I
6. Problem solving II or exposure II
7. Plan for the future
8. Booster session (after 4 week)

**Optional modules (sessions 2-7)**

1. Rumination and worries
2. Acceptance
3. Relaxation
4. Reducing alcohol
5. Self-worth
6. Perfectionism
7. Appreciation and gratefulness
8. Sleep

### Guidance

During the intervention, participants in both web-based conditions received support in the form of brief standardized emails (reminders) in the chat function of the web-based environment in case they were inactive. Participants received up to 3 weekly reminders by email. Moreover, participants in both web-based conditions could use the chat or messaging function to ask technical or user-related questions (eg, problems getting web-based access).

Participants in the computer-guided condition received automatically generated feedback messages upon completion of a module with the main aim of motivating students to carry on. In contrast, in the human-guided condition, the counselors provided detailed therapeutic feedback based on the student’s output of the modules. The counselors spent approximately 30 minutes on providing feedback per session. On average, participants received feedback on 3.9 (SD 2.0; range 0-12) days after they completed their session. The counselors were 6 female bachelor’s-level psychologists, 5 female and 1 male master’s-level psychologist, and 1 male PhD-level health care psychologist with over 10 years of prior clinical experience, who also supervised the web-based guidance and baseline intakes weekly.

### CAU Condition

Participants in the CAU condition were informed about or referred to conventional care services, both internal and external to the UvA. It should be noted that information about the available services was also provided to participants in both intervention groups. However, students in the CAU group were strongly advised to seek support. Medical health services used during the trial were monitored in all groups through self-report questions after treatment (t3), at 6-month follow-up (t4), and at 12-month follow-up (t5). Participants in the CAU group were assessed at the same time points (with the same measures) as in the 2 intervention conditions.

### Safety Monitoring

Ample attention was paid to warrant safety and decrease adverse effects during the trial. For this purpose, a *suicide protocol* was developed, describing in detail what the collaborating psychologists should do in case of an alert, which is available (only in Dutch) upon request (see Multimedia Appendix 1 for details about the safety measures). To monitor for sharp increases in complaints, the Patient Health Questionnaire (PHQ; PHQ-4) [36] was administered before each treatment session or weekly via email (CAU). Suicide risk was monitored using item 3 (“feeling down, depressed, or hopeless”) of the PHQ-4 and item 9 (“thought that you would be better off dead, or of hurting yourself”) of the Beck Depression Inventory-II [37]. All items were rated on a 0 to 3 scale. The counselors and main researchers received an automatic alert and contacted the participant by telephone if they (1) reported feeling down, depressed, or hopeless “more than half the days” or “nearly every day” (score>1) and reported having thoughts that they would be better off dead “several days” (score>0) or (2) reported having thoughts that they would be better off dead “more than half of the days” or “nearly every day” (score>1). The counselors then interviewed the participant using 6 standardized questions to rate the suicide risk and took the necessary precautions after consulting a supervisor (JK). When deemed necessary, participants were called once more, and then referred to the appropriate services. Of those allocated to 1 of the web-based intervention groups, 13.3% (71/534) had at least 1 alert and were contacted. Participants in the CAU condition were also contacted, but their data were not stored for pragmatic reasons.

### Measures

Participants who fulfilled the inclusion criteria were telephoned by appointment by a trained psychologist. The MINI [33] was administered by telephone to establish *Diagnostic and Statistical Manual of Mental Disorders* (Fourth Edition) classifications with respect to mood and anxiety disorders, bipolar disorder, psychosis, and suicidal ideation. After the interview, students were briefly informed about their complaints and whether they could be included in the study.

The primary outcome measures were the PHQ (depression; PHQ-9) and the GAD-7, which both have good psychometric properties and are often used in the context of web-based interventions. The PHQ-9 [38] is a 9-item self-report questionnaire focused on depressive symptoms experienced over the past 2-week period, such as mood, sleep, and appetite. Items are rated from 0 (not at all) to 3 (nearly every day), with total scores ranging from 0 to 27. The PHQ-9 is suited for samples at risk for depression, with high specificity (0.94) and somewhat low sensitivity (0.77) in an unselected primary care sample, which is comparable to a student sample [39]. PHQ-9 scores of 5, 10, 15, and 20 represented mild, moderate, moderately severe, and severe depression, respectively [39]. The Cronbach α value in this study was .81 at baseline. The 7-item GAD [32] is a self-report questionnaire measuring anxiety symptoms (eg, “Not being able to stop or control worrying” and “Feeling nervous, anxious, or on edge”). Items are rated from 0 (not at all) to 3 (nearly every day), with total scores ranging from 0 to 21. Mild, moderate, and severe levels of anxiety are indicated by cut-off scores of 5, 10, and 15 on the GAD-7, respectively [32]. The GAD-7 questionnaire has good psychometric properties, including a good test-retest reliability (intraclass correlation coefficient=0.83) [32] and a good internal consistency (.79<Cronbach α<.91) [40]. The Cronbach α level in this study was .84 at baseline.

Secondary outcome measures for alcohol and drug use were administered at all time points. The Drug Abuse Screening Test–10 items [41] was used to screen for drug abuse over the past 12 months. The 10 items can be answered with “yes” (score=1) or “no” (score=0), and 1 item (“Are you always able to stop abusing drugs when you want to?”) is reverse keyed. In the case where the first item (“Have you used drugs other than those required for medical reasons?”) was answered with no (0), the other items were not administered because all remaining items are concerned with problems related to drug use. The internal consistency of this scale in this study was 0.70 at baseline. Alcohol use was measured using the abbreviated Alcohol Use Disorder Identification Test [42]. This 3-item instrument assesses the quantity and frequency of drinking and binge-drinking sessions. Items are ranked from 0 to 4; total scores range from 0 to 12. Higher scores indicate more hazardous drinking. Alcohol Use Disorder Identification Test has been validated in student populations [6]. The internal consistency of this scale in this study was 0.73 at baseline.

We examined the quality of life with the subjective health item (visual analog scale) from the EQ-5D-5L at all time points except for midtreatment [43]. Medical service use was assessed with 2 items from the Treatment Inventory of Costs in Patients with Psychiatric Disorders [44] after treatment and at 6 months and 12 months. The first item includes the frequency of contact with conventional care services (eg, general practitioner, study adviser, psychologist or psychiatrist, medical specialist), and the second item includes the use of medication. We did not use this questionnaire to calculate implicit medical costs but to compare the use of medical services across treatment groups. Client satisfaction was assessed after treatment with the Client Satisfaction Questionnaire (CSQ-8) [45]. The CSQ-8 consists of 8 items (eg, “In an overall, general sense, how satisfied are you with the web-based health support you have received?”), scored on a 1 (“quite dissatisfied”) to 4 (“very satisfied”) scale, with total scores ranging between 8 and 32. A higher score on the CSQ-8 indicates a higher level of satisfaction related to the intervention. The CSQ-8 is a standardized satisfaction measure reporting very good internal consistency (Cronbach α=.83 to .93) and high validity [46]. The internal consistency of this measure was 0.93 in this study after treatment.

Finally, treatment adherence was measured by tracking the activities in Minddistrict. As outcome measures, we collected the average number of sessions completed and whether participants completed all sessions [47]. For descriptive purposes, we also extracted the duration of the treatment for those completing it and the number of times they scored above the cut-offs for a “suicide trigger.”

### Statistical Analysis

Descriptive statistics, ANOVAs (for categorical and continuous data), and *χ*^2^ tests (for categorical data only) were used to determine whether patient characteristics (sociodemographic and clinical) or use data (adherence) were similar across experimental conditions. To handle missing values for the main outcomes, multiple imputation was used. The Markov Chain algorithm was used to impute 50 data sets, with a maximum of 100 iterations for each imputation. This approach is particularly useful in case of high attrition (approximately 50%), as it minimizes the loss of statistical power when examining the relationship between variables [48]. On the basis of all 48 variables in our imputation model (including auxiliary variables), the (research) attrition rate was 48.7%. Note that recent studies have shown that the proportion of missing data should not be used as a guide for imputation per se, and when done responsibly, imputation for large amounts of missing data can still reduce bias [49]. Continuous data were imputed using predictive mean matching, while categorical variables were imputed using logistic regression. Various sociodemographic variables (eg, age, gender, and student status), the allocated experimental condition, and some auxiliary variables (eg, social anxiety, social phobia, and loneliness) were used as predictors only. In addition, all main outcomes of this study were used as predictors as well as values to be imputed. In the main analyses, the results were pooled across the 50 imputed data sets using the rules to account for the uncertainty introduced by the imputation process by Rubin [50]. Our a priori power calculations were based on the mixed factors 3×3 interaction difference between the 3 treatment arms and 3 time points (baseline, midtreatment, and after treatment), and taking a dropout rate of 35% into account, as well as a correlation between repeated measures of *r*=.50. A total sample size of 276 (92 in each group) would be needed to estimate a small within-between interaction effect [11]. With a corrected *P* value of .025 based on 2 primary outcomes, the total sample size required would be 369, which is amply exceeded by our sample size of 801.

Linear mixed models were used to estimate treatment effects over time. We included a fixed between-subjects effect for intervention group and a fixed within-subjects effect for time (ie, assessments at 3 or 5 sequential time points, respectively), as well as their 2-way interaction. We modeled linear, quadratic, or asymptotic effects of time by, including first or flattened second-degree polynomials created with the poly function in R statistical software (R Foundation for Statistical Computing) [51] and compared their fit. We only interpreted results from the best-fitting model. This was a convex pattern for complaints (depression, anxiety, and drug and alcohol use) and a concave pattern for health concerning the long-term outcomes, and a linear pattern for the short-term primary outcomes. As we compared 3 groups in the main analyses, we coded condition as a factor with 3 levels. We also estimated a random intercept to allow for individual variations in the intercepts for each subject. We imposed an autoregressive structure to the residual covariance matrix to correct for autoregression of repeated assessments within the same subject. It assumes that correlations between any 2 elements are equal to (Pearson) *r* for adjacent elements, *r^2^* for 2 elements separated by a third, and so on. Maximum likelihood was used as the overall estimation method. Plots were made using the Grammar of Graphics framework (*ggplot*) in R statistical software.

For the primary outcomes, both short-term (t3) and long-term (t4 and t5) analyses were performed based on the 3 conditions, using CAU as the reference group and subsequently computer-guided iCBT as the reference group (for pairwise comparisons). For the secondary outcomes, only long-term outcomes were analyzed (t5). Bonferroni corrections were applied to all exploratory multilevel models to control for type I error related to multiple testing, while avoiding an increased risk for type II error. Thus, based on approximately 30 tests, corrected *P* values ≤.002 were considered statistically significant. *P* values between .002 and .025 were considered marginally significant. Effect sizes were calculated for marginally significant interactions, transforming *t* values to Pearson *r* [52]. For all main analyses, SPSS (version 28.0.1.0; IBM Inc) [53] was used.

## Results

### Random Allocation and Characteristics of Participants

**Figure 1:**
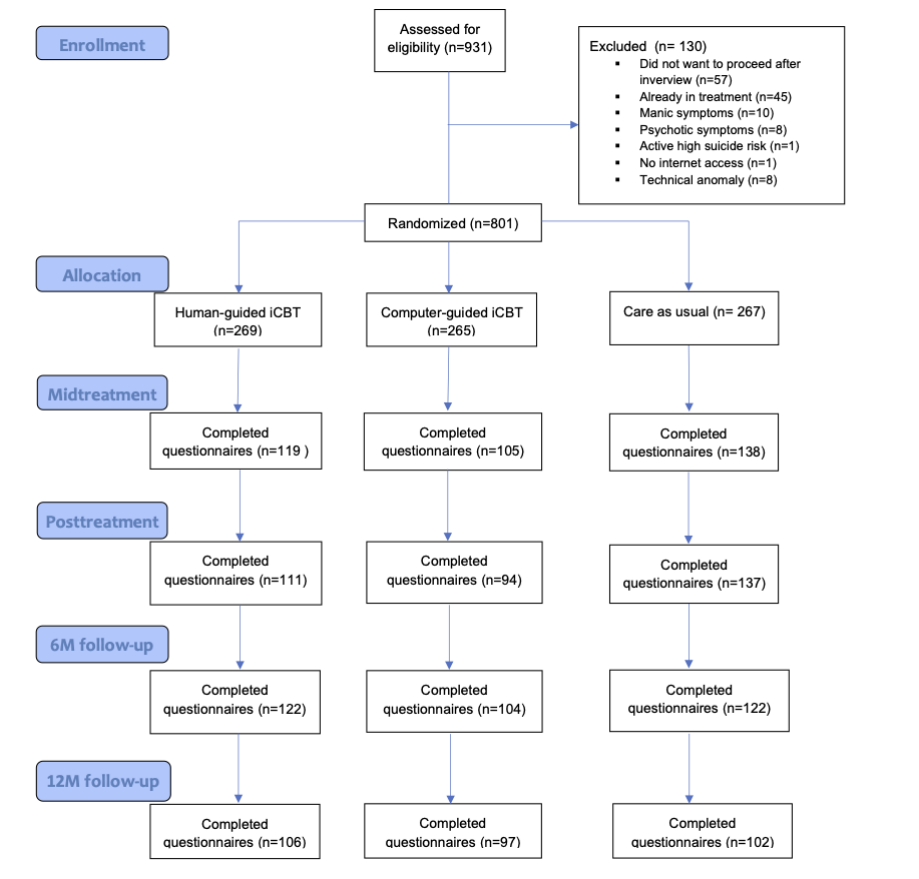
Flow diagram illustrating the progression of participants through the stages of the study. iCBT: internet-based cognitive behavioral therapy.

A total of 801 participants were eligible and thus randomized across conditions: 269 were allocated to the human-guided group, 265 to the computer-guided group, and 267 to CAU group (Figure 1). The average age of participants was 23.9 (SD 4.6, range 17-55) years; 71.5% (573/801) were female, and 7.5% (6/801) of the participants indicated that their gender was *Other*. The sample contained 387 (48.3%) undergraduate students (mean age 21.6, SD 3.6 y; range 17-55 y), 315 (39.3%) master’s students (mean age 25.5, SD 3.9 y; range 18-53 y), and 89 (11.1%) PhD students (mean age 29.4, SD 4.8 y; range 24-51 y).

The average level of alcohol use (mean 3.72, SD 2.09) was below the average for students and far below the cut-off for harmful drinking [6]. At baseline, the average number of classifications according to the MINI was 1.79 (SD 1.74). The most common diagnosis was a current depressive episode (287/801, 35.8%), followed by generalized anxiety disorder (154/801, 19.2%). Around half of the sample had either no classification (222/801, 27.7%) or 1 potential disorder (219/801, 27.3%); the other half (360/801, 45%) had more than 1 potential disorder. For an overview of diagnostic classifications, see Table 1.

**Table 1:** Diagnostic and Statistical Manual of Mental Disorders (Fourth Edition) classifications based on the Mini-International Neuropsychiatric Interview administered at baseline (N=801).

			Current, n (%)		Lifetime, n (%)
**Classifications**					
	Depression	287 (35.8)		199 (24.8)	
	Dysthymic disorder	36 (4.5)		N/A^a^	
	Manic episode	N/A^b^		11 (1.4)	
	Hypomanic episode	4 (0.5)		43 (5.4)	
	Panic disorder	36 (4.5)		92 (11.5)^c^	
	Agoraphobia	65 (8.1)		N/A	
	Social phobia	149 (18.6)		N/A	
	Simple phobia	17 (2.1)^d^		N/A	
	Obsessive-compulsive disorder	46 (5.7)^e^		N/A	
	Post-traumatic stress disorder	27 (3.4)^f^		N/A	
	Generalized anxiety disorder	154 (19.2)		N/A	
	Mixed anxiety-depressive disorder	85 (10.6)^g^		N/A	
	Psychotic syndrome	N/A^b^		16 (2)	
**Comorbidity**					
	No classification	222 (27.7)		N/A	
	1 disorder	219 (27.3)		N/A	
	2 disorders	111 (13.9)		N/A	
	3 disorders	114 (14.2)		N/A	
	>3 disorders	135 (16.9)		N/A	
**Suicide risk**					
	Low	130 (16.2)		N/A	
	Moderate	59 (7.4)		N/A	
	High	19 (2.4)		N/A	
	No	591 (73.8)		N/A	

^a^N/A: not applicable.

^b^This was an exclusion criterion for the study.

^c^Based on a lower number of participants (n=726).

^d^Based on a lower number of participants (n=713).

^e^Based on a lower number of participants (n=770).

^f^Based on a lower number of participants (n=771).

^g^Based on a lower number of participants (n=717).

### Adherence and Satisfaction

For the human-guided condition, the largest groups were composed of those doing either 0 sessions (63/268, 23.5%) or 8 sessions (48/268, 17.9%). For the computer-guided condition, the most common number of sessions completed was also 0 (81/264, 30.7%), followed by 1 (53/264, 20.1%). The average number of sessions completed in the human-guided condition was 3.32 (SD 3.02; median 2, IQR 6); in the computer-guided condition, this was 2.54 (SD 2.75; median 1, IQR 4; *F*_1_=9.85; *P*=.002). The completion rate (defined as 7 or 8 sessions done) for the human-guided condition was also significantly higher than that for the computer-guided condition: 26.9% (72/268) versus 15.5% (41/264) cases completed treatment (n=532; χ^2^_1_=10.2; *P*=.001). Finally, participants in both the human-guided and computer-guided conditions were significantly more satisfied after treatment compared with those in the CAU condition (*F*_2_=45.2; *P*<.001).

### Optional Modules and Psychological Care

Only those participants who completed more than 1 session were enabled to choose optional modules (289/534, 54.1%). Among those participants, a total of 28.5% (83/291) completed 1 optional module, 21.6% (63/291) completed 2 modules, and 49.8% (145/291) completed 3 to 6 modules (median 2, IQR 3). There were no significant differences between the iCBT conditions in terms of optional modules made (*F*_1_=0.74; *P*=.39). Of those enabled to choose an optional module, 13.7% (40/291) of participants chose the optional module for alcohol use-related problems. Asked retrospectively at 6 months, participants in the CAU condition reported to have visited a psychologist or psychiatrist more often (65/265, 24.5%) during the 6 months prior than the participants in the human-guided (38/269, 14.1%) or computer-guided (49/267, 18.3%) conditions (n=795; χ*^2^*_2_=7.96; *P*=.02).

### Efficacy Over Time

**Figure 2:**
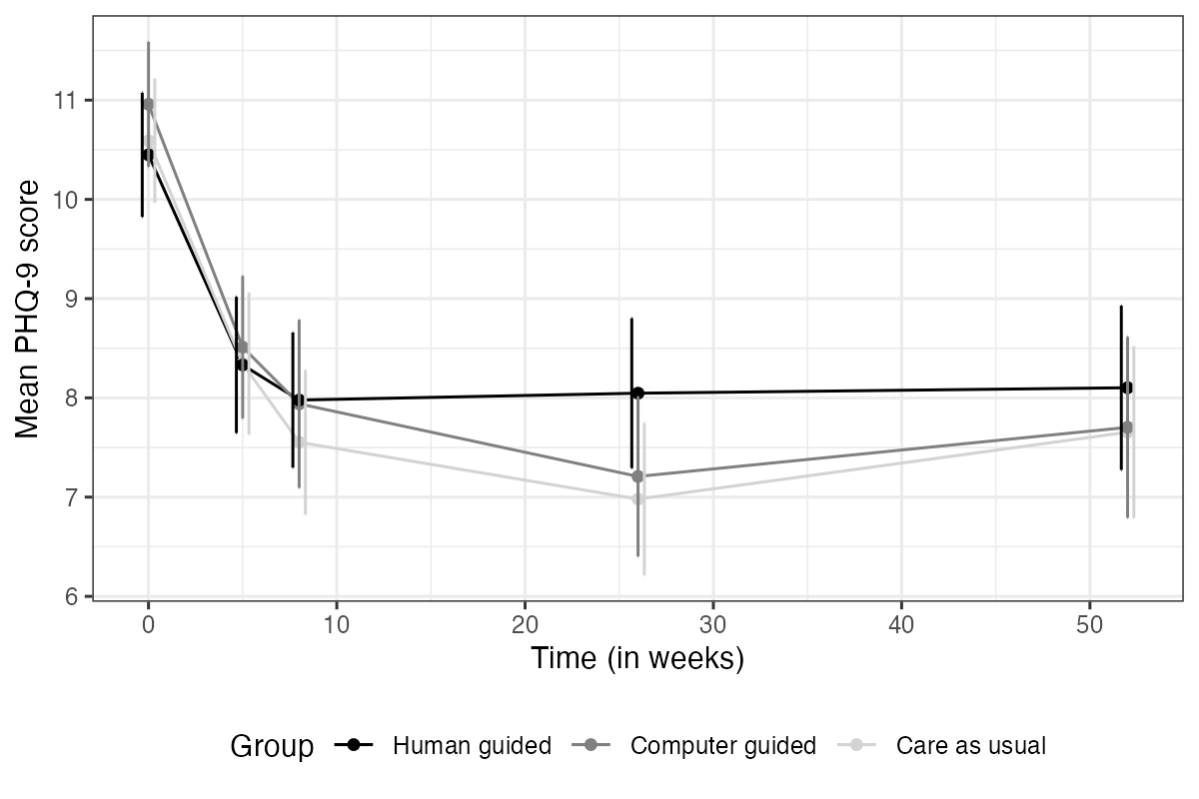
Mean depression over time by condition. PHQ-9: Patient Health Questionnaire–9 Note. Vertical lines represent error bars (with a 95% confidence interval); non-overlapping error bars indicate that the true means are likely to be different from each other.

**Figure 3:**
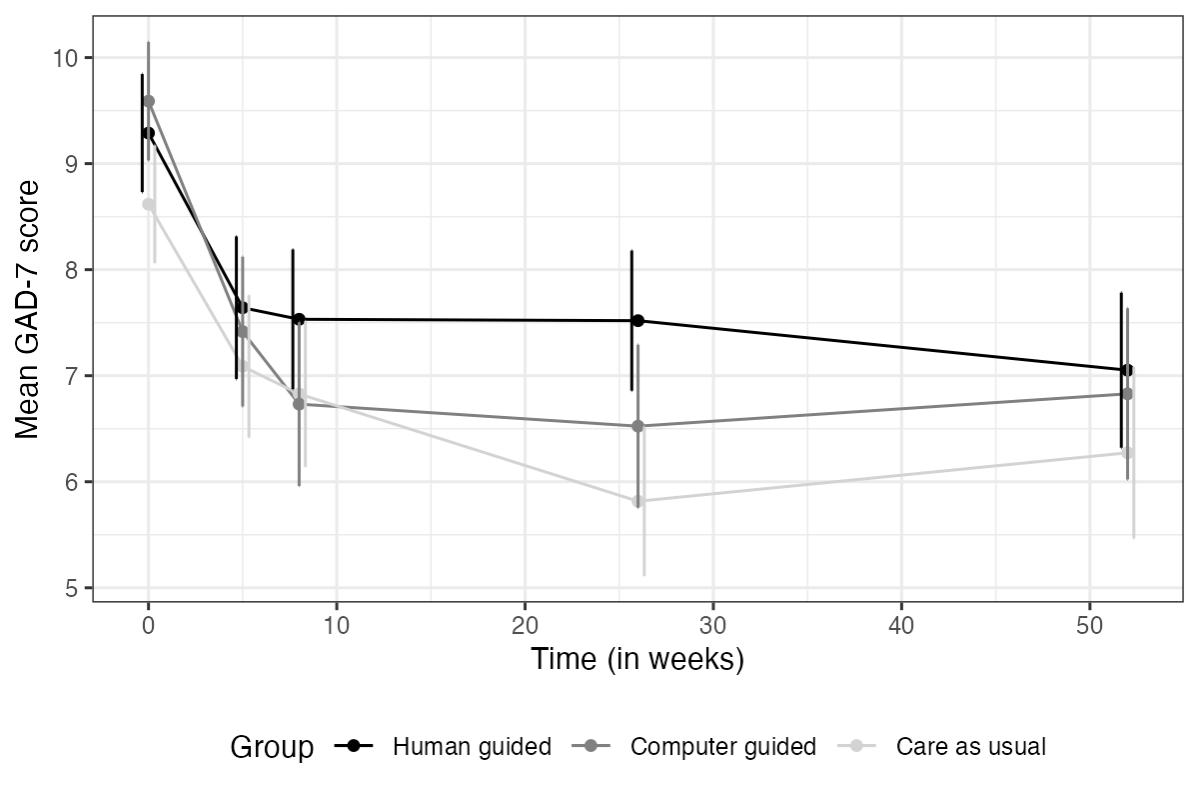
Mean anxiety over time by condition. GAD-7: Generalized Anxiety Disorder scale–7 items. Note. Vertical lines represent error bars (with a 95% confidence interval); non-overlapping error bars indicate that the true means are likely to be different from each other.

For the course of symptoms over time across the 3 conditions, see Figure 2 (depression) and Figure 3 (anxiety). For the estimated marginal means of the 2 primary outcome measures across treatment groups at each assessment point, see Tables 2 and 3.

**Table 2:** Estimated marginal means and SEs for the primary outcome measures at all assessment points for the 3 conditions—depression (Patient Health Questionnaire–9).

Condition	Time				
	Baseline, mean (SE)	Midtreatment, mean (SE)	After treatment, mean (SE)	6 months, mean (SE)	12 months, mean (SE)
Human guided	10.591 (0.318)	8.345 (0.363)	7.553 (0.371)	6.980 (0.391)	7.654 (0.441)
Computer guided	10.958 (0.321)	8.511 (0.366)	7.940 (0.432)	7.208 (0.411)	7.703 (0.465)
Care as usual	10.449 (0.319)	8.332 (0.350)	7.978 (0.347)	8.047 (0.385)	8.102 (0.422)

**Table 3:** Estimated marginal means and SEs for the primary outcome measures at all assessment points for the 3 conditions—anxiety (Generalized Anxiety Disorder scale–7 items).

Condition	Time				
	Baseline, mean (SE)	Midtreatment, mean (SE)	After treatment, mean (SE)	6 months, mean (SE)	12 months, mean (SE)
Human guided	8.617 (0.283)	7.088 (0.342)	6.828 (0.350)	5.816 (0.359)	6.273 (0.412)
Computer guided	9.589 (0.285)	7.416 (0.360)	6.732 (0.394)	6.524 (0.392)	6.829 (0.412)
Care as usual	9.288 (0.284)	7.641 (0.343)	7.532 (0.336)	7.519 (0.337)	7.052 (0.373)

### Short-Term Efficacy (t3)

According to our preregistered protocol, we analyzed the efficacy over time until after treatment (t3) for the 2 primary outcome measures. For depression, no significant time×group interaction was observed when comparing both the human-guided (B=.08, 95% CI –.05 to .21; *P*=.25) and the computer-guided (B=.06, 95% CI –.09 to .21; *P*=.43) conditions to CAU. For the direct comparison between human-guided and computer-guided iCBT, no significant time by treatment interaction was found (B=.02, 95% CI –.14 to .17; *P*=.86).

For anxiety, none of the interventions differed significantly from CAU over time: *human-guided versus CAU* (B=.01, 95% CI −.11 to .13; *P*=.89) and *computer-guided iCBT versus CAU* (B=.13, 95% CI –.003 to .27; *P*=.05). No significant differences were found when comparing human-guided and computer-guided iCBT directly with each other (B=−.12, 95% CI –.26 to .01; *P*=.08).

### Long-Term Efficacy for the Primary Outcomes (t4)

Next, we analyzed the primary outcomes until t4 (6 months) follow-up. Over 6 months, no significant time by condition interactions were found for either depression or anxiety, indicating that the slopes for the 3 conditions were similar. Some comparisons were marginally significant (see Tables 4 and 5 for coefficients). Next, we added the covariate of “psychological care” to control for this higher level of care in the CAU condition. For both depression and anxiety, results remained marginally significant for both intervention conditions compared with CAU yet did not reach the corrected *P* value of .002. For depression, human-guided iCBT was marginally more efficacious than CAU (B=3.21, 95% CI 0.54-5.89; *P*=.02; *r*=0.08). The computer-guided condition was also marginally more efficacious compared with CAU (B=3.33, 95% CI 0.61-6.05; *P*=.02; *r*=0.08). For anxiety, similar results were obtained: *human-guided* iCBT *versus CAU* (B=3.08, 95% CI 0.77-5.40; *P*=.01; *r*=0.09) and *computer-guided iCBT versus CAU* (B=3.12, 95% CI 0.62-5.62; *P*=.02; *r*=0.08).

**Table 4:** Long-term (6-month) results of linear mixed model for pairwise comparisons of conditions (depression).

Fixed effects	Comparison						
	Human guided vs care as usual		Computer guided vs care as usual			Human guided vs computer guided	
	Β (SE)	*P* value	Β (SE)	*P* value	Β (SE)		*P* value
Intercept	*8.22* ^a^ *(0.29)*	*<.001*	*8.22 (0.29)*	*<.001*	*7.66 (0.30)*		*<.001*
Time	*4.49 (0.97)*	*<.001*	*4.49 (0.97)*	*<.001*	*7.39 (0.98)*		*<.001*
Condition	−0.56 (0.41)	.18	−0.30 (0.44)	.50	−0.26 (0.43)		.55
Time×condition	2.91 (1.36)	.03	3.15 (1.38)	.02	−0.25 (1.39)		.86

^a^Italicized values are statistically significant (*P*<.05).

**Table 5:** Long-term (6-month) results of linear mixed model for pairwise comparisons of conditions (anxiety).

Fixed effects	Comparison							
	Human guided vs care as usual			Computer guided vs care as usual			Human guided vs computer guided	
	Β (SE)	*P* value	Β (SE)		*P* value	Β (SE)		*P* value
Intercept	*7.72* ^a^ *(0.25)*	*<.001*	*7.72 (0.25)*		*<.001*	*6.59 (0.27)*		*<.001*
Time	*3.40 (0.83)*	*<.001*	*3.40 (0.83)*		*<.001*	*6.01 (0.88)*		*<.001*
Condition	−1.13 (0.36)	.002	−0.65 (0.41)		.11	−0.48 (0.40)		.23
Time×condition	2.61 (1.15)	.02	2.84 (1.27)		.03	−0.24 (1.29)		.85

^a^Italicized values are statistically significant (*P*<.05).

### Long-Term Efficacy for the Primary and Secondary Outcomes (t5)

**Figure 4:**
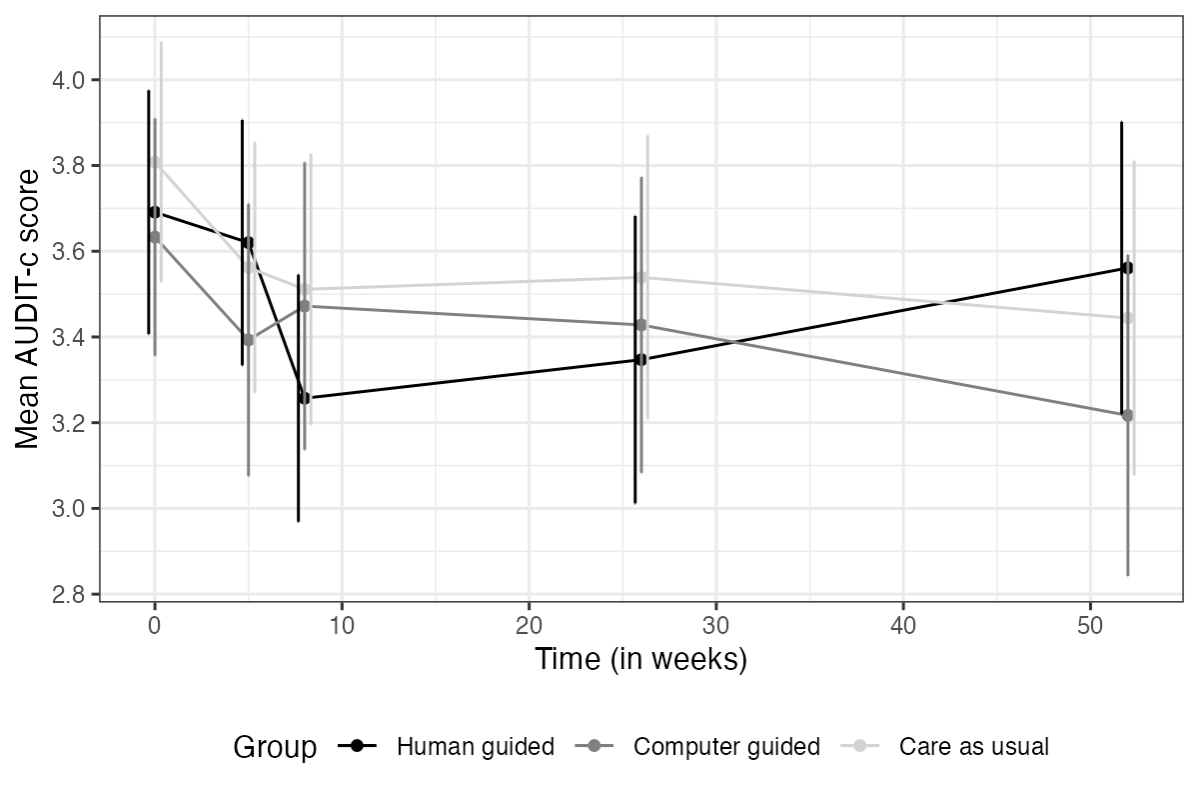
Alcohol use over time. AUDIT-C: Alcohol Use Disorder Identification Test alcohol consumption questions. Note. Vertical lines represent error bars (with a 95% confidence interval); non-overlapping error bars indicate that the true means are likely to be different from each other.

**Figure 5:**
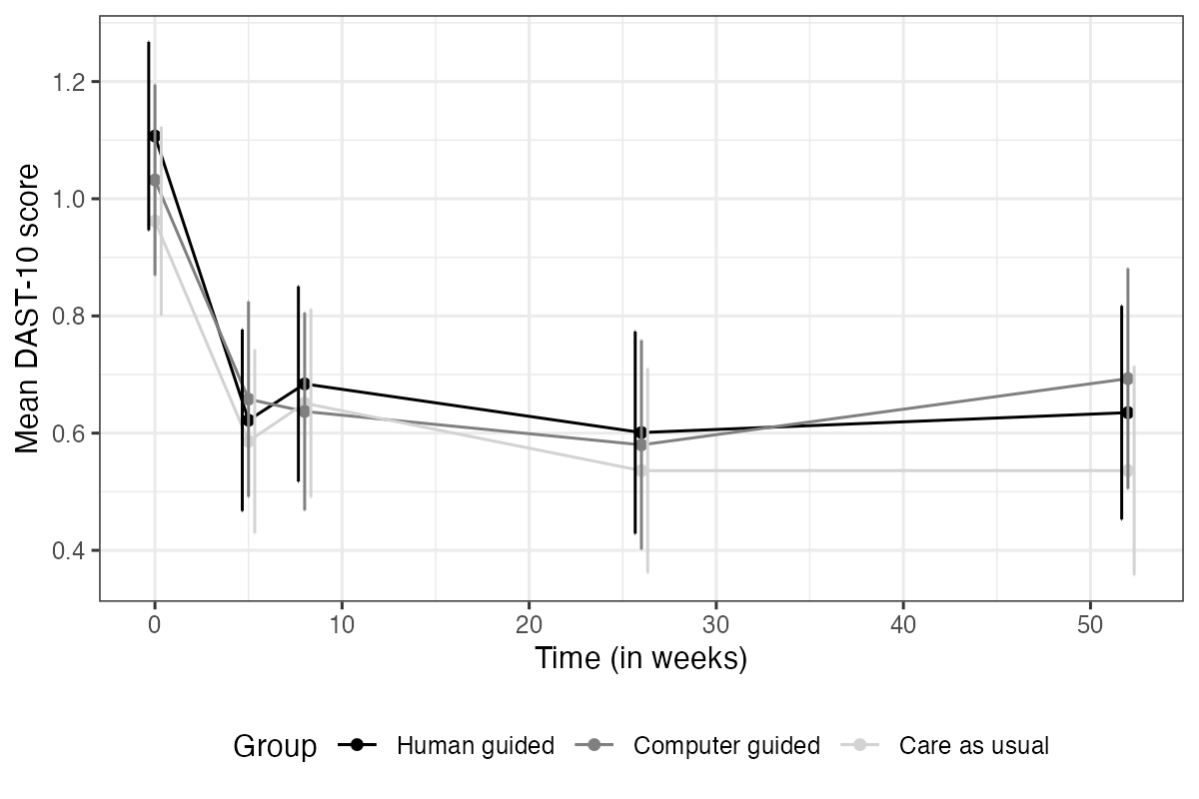
Drug use over time. DAST-10: Drug Abuse Screening Test–10 items. Note. Vertical lines represent error bars (with a 95% confidence interval); non-overlapping error bars indicate that the true means are likely to be different from each other.

**Figure 6:**
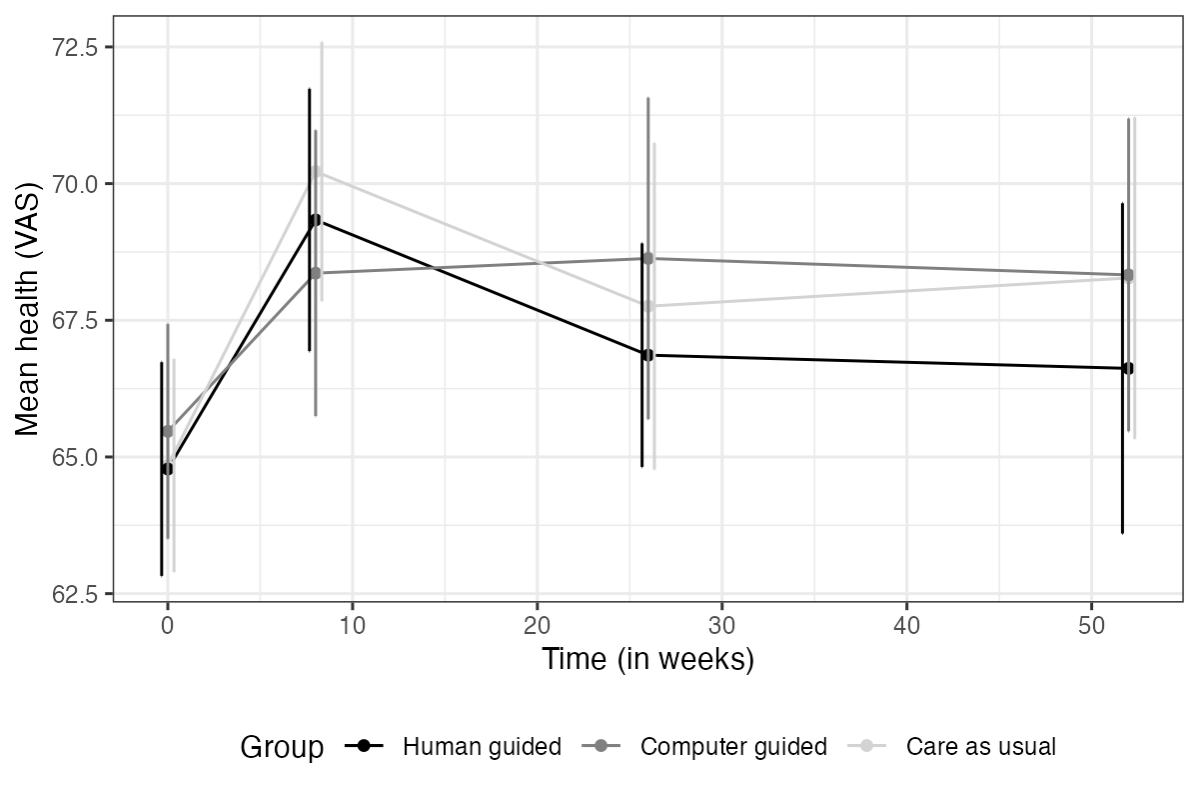
Subjective health over time. VAS: visual analog scale. Note. Vertical lines represent error bars (with a 95% confidence interval); non-overlapping error bars indicate that the true means are likely to be different from each other.

All material related to the outcomes at t5 can be found in Multimedia Appendix 2 for the primary outcomes and Multimedia Appendix 3 for the secondary outcomes. In summary, none of the interactions between time and treatment conditions were significant. However, time effects for depression, anxiety, and drug use were highly significant, indicating that all groups improved over time (*P*<.001). The time effects for alcohol use and subjective health were not significant (*P*=.08 and .30, respectively). Figures for the secondary measures are shown in Figures 4-6.

### Sensitivity Analyses (t4)

To add further validity to our results, we included 2 additional sensitivity analyses (see Multimedia Appendix 4 for details). First, we reran analyses on the groups that did at least 1 session (655/801, 81.8%), excluding those who did 0 sessions. The intent of this analysis was to assess whether those with at least some adherence to treatment, regardless of completed assessments, had a similar outcome compared to the whole group (this is an imputed data set). For both depression and anxiety, human-guided and computer-guided iCBT were marginally more efficacious than CAU (.02<*P*<.05; 0.07<*r*<0.09).

Second, we reran the analyses for the original data set without imputation. This latter data set contained all subjects, yet most of them had at least 1 missing value which was not corrected for. With this analysis, we examined a potential difference between the imputed and the unimputed data set, which may indicate a bias. For both depression and anxiety, human-guided and computer-guided iCBT were significantly more efficacious than CAU (*P*<.001; 0.11<*r*<0.16).

## Discussion

### Principal Findings

In this study, we compared 2 types of web-based interventions with CAU, both regarding the short and long-term outcomes in a large sample of university students with elevated levels of depression and anxiety symptoms. Overall, reductions in anxiety and depression were seen in all 3 conditions over time, yet no significant differences between conditions were observed over time.

In the CAU group, approximately one-quarter (65/265, 24.5%) of participants sought care from a mental health care professional during the intervention period. When controlling for this psychological care in the CAU condition, both iCBT conditions were marginally more efficacious compared with CAU across the 6-month period. This implies that iCBT is a viable alternative for CAU in a large educational setting. It seems that most students found the help they needed, or at least felt reassured to know that help was available.

No significant differences in symptoms were found in the direct comparison between the human-guided intervention and computer-guided intervention. However, as expected, the difference between human-guided and computer-guided was present for the adherence measures; students in the human-guided condition completed more sessions and subsequently did more optional modules that focused on specific problem areas. It could be that human guidance encouraged students to persist with the intervention. However, higher adherence to the human-guided intervention was not necessarily associated with larger benefits, as indicated by our analyses of symptoms. This could be due to the small yet significant difference in adherence between the conditions (approximately 1 session), which may be too small to yield an observable and clinically relevant difference. Future studies could test the hypothesis whether human guidance indirectly impacts symptom outcomes through its effect on adherence (ie, mediation analysis).

### Comparison to Previous Studies

In line with Karyotaki et al [10], both web-based interventions did not outperform the CAU condition. Our finding replicates and extends this previous finding, as our study included more students and students with higher levels of anxiety and depression. Some studies suggested that therapist-guided iCBT is more efficacious for individuals with elevated levels of anxiety or depression [25,27], but this assumption was not supported by our findings. The fact that the interventions were not more efficacious than CAU could have several reasons. First, it could be considered that our pattern of findings reflects regression to the mean, which is typical in longitudinal studies in which participants are selected on the basis of elevated scores at baseline [54]. Any potential effect of the interventions may have been overshadowed by the effect of this statistical artifact, which would have been present in all conditions. Second, although comparable to other web-based intervention studies, the adherence rates in this study were rather low, resulting in a potential loss of efficacy. Only a quarter (72/268, 26.9%) of the participants completed the human-guided intervention. In the computer-guided condition, only half (130/264, 49.2%) of the participants did ≥2 sessions. The attrition rates are generally high in internet interventions (especially in so-called unguided formats), and this should be improved in future trials [28]. A third explanation could be that students who do not seek help on their own initiative may have a diminished treatment response, due to factors of decreased intrinsic motivation, low awareness of their illness burden, and unrealistic expectations about the time investment required.

The lack of any significant differences between human-guided and computer-guided conditions (*P* values>.025 as per preregistration) is not in line with previous findings from a meta-analysis showing that human guidance is slightly yet significantly more efficacious than *technological guidance* [28], at least in the short run, and begs further explanation. It could be a result of the fact that the intensity of guidance in both conditions was quite similar. Although students in the human-guided condition received weekly personalized feedback from psychologists, students in the computer-guided condition received a considerable amount of support as well, including automated feedback, regular reminders sent by a psychologist, personalized responses to queries in the chat, and phone calls in case of suicide alerts. Of those who were actively engaged on the eHealth platform, around 13.3% (71/534) received such phone calls. This may have led to the impression among students in the computer-guided condition that they did receive personalized support and that they perceived a sense of *therapeutic presence*, that is, someone in the outside world that had their concerns in mind. An alternative reason could be that psychologists in the human-guided condition, although they received supervision, were not specifically trained for this intervention and they were not delivering therapy in a strict sense, but rather some form of support, which may have been experienced as disappointing for some students, who expected more e-therapy, as qualitative interviews after the trial suggested (JA Koelen, unpublished data, April 2024). Third, students in the computer-guided condition received immediate, automatized feedback, whereas human guidance was delivered after relatively long intervals between sessions, which took an average of 3.9 (SD 2.0) days. Further research should aim to clarify to what extent these factors contribute to varieties in effectiveness. A final explanation could be that the differences between *guided* and *unguided* web-based interventions (often iCBT) reported in the literature refer to short-term outcomes, as emerging evidence indicates that the differences may be short-lived [27].

The lack of a significant difference between the human-guided and computer-guided conditions also raises questions concerning the working mechanisms of web-based therapy [55]. As with regular therapy, it is still unclear whether web-based therapy works through mechanisms common to all therapies (eg, the therapeutic alliance, the mobilization of hope), or those specific to certain types of therapies (eg, cognitive restructuring in CBT), or both. Notably, technical components of iCBT (ie, specific mechanisms) were present in both conditions, and the nonexistent difference between conditions would plea for the effectiveness of such components. Previous qualitative analyses of emails from web-based counselors providing iCBT suggested that interventions that were regarded as most helpful (and were associated with better outcomes) were best classified as *common factors* [56,57]. These interventions included validation of completed exercises, anticipation of pending assignments (ie, changing expectations of personal effectiveness), as well as empathy and self-disclosure of web-based counselors. Some of these interventions were also provided with the automated feedback in the computer-guided condition, which could also explain the absence of significant differences between conditions.

### Strengths and Limitations

A major strength of this trial, first, is that we compared a human-guided and a computer-guided form of iCBT head-to-head, and to an active control group. Second, our sample was relatively large, enabling us to detect small interaction effects. Third, we included students with elevated levels of symptoms, with nearly three quarters of the students (579/801, 72.3%) presenting with at least one potential mental disorder, which makes this a representative sample of students in terms of burden of disease [3]. Finally, we assessed the long-term outcome of our interventions.

Several limitations need to be considered when interpreting the results of this study. First, nearly all participants were included during the COVID-19 pandemic, which reached its first peak in the Netherlands in May 2020. More precisely, only 1.9% (15/801) of the participants completed the entire study before the onset of the COVID-19 pandemic. Intervention effects may have been distorted due to this crisis, for instance, due to an increase in symptoms during the trial [34]. Second, the number of PhD students included in this study was relatively low, which needs to be considered when extrapolating our findings to US student samples. This small number is mainly attributable to the clear distinction between master’s and PhD students that is made in the Netherlands, where a Master of Science degree is the typical end point of study and only few students are selected to do a PhD trajectory. Third, as expressed by some students, the modules were *text heavy* and lacked interactive functionalities, which may have diminished its attractiveness, so this could be improved. Yet, the intervention had been adapted especially for students, and the text was enriched by elements of persuasive design, which are known to increase adherence, such as creating an overview, use of metaphors, and creating a sense of control and purpose [58]. Fourth, in hindsight, we might have recruited more psychologists for guidance, which would have resulted in swifter feedback, although it is debatable whether swifter feedback results in greater efficacy [59]. Fifth, the relatively large amount of missing data could have introduced a bias in our data. However, we limited that risk of bias by using the Markov Chain algorithm, which is adept at capturing data dependencies, and maintaining data set integrity [60]. A recent meta-analysis [28] of web-based interventions found an average of 48% attrition for human-guided interventions and an even higher percentage (51%) for those using technological guidance only. These figures are comparable to those reported in this study. Nevertheless, a major interest of future studies should be to decrease dropout. Sixth, future research could aim to clarify the optimal length and intensity for web-based treatments, as we followed a classic format with weekly session. Some studies of regular (face-to-face) therapy have shown, for example, that a more concentrated approach (ie, an equal number of sessions with increasing frequency) can bring about more optimal outcomes [61]. Seventh, some studies have shown that rewarding students for completing questionnaires, as was done in this trial, could lead to an underestimation of intervention effects [20]. Eighth, we could not establish the effect of sessions made irrespective of the intervention type because the number of sessions completed was contingent upon the allocated condition. This requires another type of experimental research. Ninth, future studies should pay more attention to individual differences and moderators of outcome. Finally, we assessed the outcome only in terms of symptoms, and not in terms of underlying vulnerabilities (eg, elevated levels of negative affect), which would have suited a transdiagnostic approach.

### Conclusions

In conclusion, the results of this study indicated both iCBT interventions were not inferior to CAU (which did include the possibility to visit other mental health care professionals, who had a limited capacity). Moreover, we found that 1 type of iCBT (human-guided) was not more efficacious than the other (computer-guided). We conclude that iCBT offers a feasible and viable alternative for the regular health services in a university setting, which are often hard to access for students. Future studies should also examine cost-effectiveness to determine whether the additional investment in web-based psychologists is worthwhile.

## Appendix

Multimedia Appendix 1 Safety measures.

Multimedia Appendix 2 Long-term results (t5) for the primary outcomes.

Multimedia Appendix 3 Long-term results (t5) for the secondary outcomes.

Multimedia Appendix 4 Sensitivity analyses.

Multimedia Appendix 5 CONSORT checklist.

## Abbreviations

CAU: care as usual
CBT: cognitive behavioral therapy
CSQ-8: Client Satisfaction Questionnaire
GAD-7: Generalized Anxiety Disorder scale–7 items
iCBT: internet-based cognitive behavioral therapy
MINI: Mini International Neuropsychiatric Interview
PHQ: Patient Health Questionnaire
UvA: University of Amsterdam
