# Supplementary material for: Web-Based, Human-Guided, or Computer-Guided Transdiagnostic Cognitive Behavioral Therapy in University Students With Anxiety and Depression: Randomized Controlled Trial
Source: JMIR Ment Health. 2024 Jun 19;11:e50503. doi: 10.2196/50503 (PMC11222767; doi:10.2196/50503)
Supplement: Multimedia Appendix 1 [file mental_v11i1e50503_app1.pdf]

ONLINE SUPPLEMENT 1

Precautions to prevent or handle adversity ("safety monitoring")

Several measures were taken to decrease potential risks and to handle adverse effects:

1) We have developed an online tool to screen for suicidal ideation by adding a mandatory question about this in each session of the online intervention (for students in the CAU condition, these questions were sent to them separately). In deciding the cut-offs for this tool, we took a cautious approach, because we did not want to miss any signs and set the threshold to indicate any potential risk relatively low.
2) We developed a "suicide protocol" (available upon request), describing in detail what the collaborating psychologists should do in case of an alert. For example, psychologists called the students several times in case of an alert. In our experience, most of these alerts were indeed "false alerts", confirming our tendency to err on the side of caution. Of course, we still could have missed sings of serious mental health problems due to stigma, but to the best of our knowledge, there have been no suicidal events associated with this trial.
3) Students were always free to quit the intervention and seek for help elsewhere. Each student received an information brochure where to find help along with the invitation to participate in this study. It is very difficult to fully eliminate the stigma around mental health problems. With this study, we think we actually reduced the stigma by lowering the threshold to seek help online.
