# Supplementary material for: Web-Based, Human-Guided, or Computer-Guided Transdiagnostic Cognitive Behavioral Therapy in University Students With Anxiety and Depression: Randomized Controlled Trial
Source: JMIR Ment Health. 2024 Jun 19;11:e50503. doi: 10.2196/50503 (PMC11222767; doi:10.2196/50503)
Supplement: Multimedia Appendix 2 [file mental_v11i1e50503_app2.pdf]

Tables S1 + S2 Long-term (12 months) results of linear mixed models for pairwise comparisons of treatment groups for the primary outcome measures

Table S1 depression (PHQ-9)

**Fixed Effects**	**Comparison**					
	Human guided versus care as usual		Computer guided versus care as usual		Human guided versus computer guided	
**Parameter**	**B (SE)**	***P* value**	**B (SE)**	***P* value**	**B (SE)**	***P* value**
Intercept	*8.34 (0.27)*	*<.001*	*8.34 (0.27)*	*<.001*	*7.90 (0.28)*	*<.001*
Time	*4.03 (0.87)*	*<.001*	*4.03 (0.87)*	*<.001*	*6.30 (0.83)*	*<.001*
Condition	-0.44 (0.38)	.25	-0.22 (0.40)	.57	-0.22 (0.40)	.58
Timexcondition	2.27 (1.20)	.06	2.71 (1.22)	.03	-0.44 (1.15)	.70

Note. Italicized values are statistically significant (P<.002)

Table S2 anxiety (GAD-7)

**Fixed Effects**	**Comparison**					
	Human guided versus care as usual		Computer guided versus care as usual		Human guided versus computer guided	
**Parameter**	**B (SE)**	***P* value**	**B (SE)**	***P* value**	**B (SE)**	***P* value**
Intercept	*7.67 (0.24)*	*<.001*	*7.67 (0.24)*	*<.001*	*6.72 (0.25)*	*<.001*
Time	*3.66 (0.74)*	*<.001*	*3.66 (0.74)*	*<.001*	*5.37 (0.79)*	*<.001*
Condition	-0.95 (0.34)	<.01	-0.46 (0.36)	.20	-0.49 (0.36)	.17
Timexcondition	1.71 (1.06)	.11	1.88 (1.09)	.09	-0.17 (1.10)	.88

Note. Italicized values are statistically significant (P<.002)
