# Supplementary material for: Web-Based, Human-Guided, or Computer-Guided Transdiagnostic Cognitive Behavioral Therapy in University Students With Anxiety and Depression: Randomized Controlled Trial
Source: JMIR Ment Health. 2024 Jun 19;11:e50503. doi: 10.2196/50503 (PMC11222767; doi:10.2196/50503)
Supplement: Multimedia Appendix 3 [file mental_v11i1e50503_app3.pdf]

Tables S1-S3 (Descriptives) and S4-S6 (Analyses) for secondary outcomes

Tables S1-S3 Estimated marginal means and standard errors for the secondary outcome measures at all assessment points for the three treatment conditions (for the primary measures, see main paper)

Table S1 alcohol use (AUDIT-C)

	Baseline	midtreatment	posttreatment	6 months	12 months
**Condition**	**Mean (SE)**	**Mean (SE)**	**Mean (SE)**	**Mean (SE)**	**Mean (SE)**
Human guided	3.81 (0.14)	3.56 (0.15)	3.51 (0.16)	3.54 (0.17)	3.44 (0.19)
Computer guided	3.63 (0.14)	3.40 (0.16)	3.47 (0.17)	3.43 (0.18)	3.22 (0.19)
Care as usual	3.70 (0.15)	3.62 (0.15)	3.26 (0.15)	3.35 (0.17)	3.56 (0.17)

Table S2 drug use (DAST-10)

	Baseline	midtreatment	posttreatment	6 months	12 months
**Condition**	**Mean (SE)**	**Mean (SE)**	**Mean (SE)**	**Mean (SE)**	**Mean (SE)**
Human guided	0.96 (0.08)	0.58 (0.08)	0.65 (0.08)	0.54 (0.09)	0.54 (0.09)
Computer guided	1.03 (0.08)	0.66 (0.09)	0.64 (0.09)	0.58 (0.09)	0.69 (0.10)
Care as usual	1.11 (0.08)	0.62 (0.08)	0.68 (0.09)	0.60 (0.09)	0.64 (0.09)

Table S3 subjective health (VAS)

	Baseline	posttreatment	6 months	12 months
**Condition**	**Mean (SE)**	**Mean (SE)**	**Mean (SE)**	**Mean (SE)**
Human guided	64.88 (0.99)	70.22 (1.21)	67.76 (1.52)	68.27 (1.50)
Computer guided	65.47 (1.00)	68.36 (1.33)	68.63 (1.50)	68.33 (1.46)
Care as usual	64.78 (1.00)	69.34 (1.22)	66.86 (1.04)	66.62 (1.54)

Note. AUDIT-C = Alcohol Use Disorders Identification Test; DAST-10 = Drug Abuse Screening Test

Tables S4-S6 Long-term (12 months) results of linear mixed models for pairwise comparisons of treatment groups for the secondary outcome measures

Table S4 alcohol use (AUDIT-C)

**Fixed Effects**	**Comparison**					
	Human guided versus care as usual		Computer guided versus care as usual		Human guided versus computer guided	
**Parameter**	**B (SE)**	***P* value**	**B (SE)**	***P* value**	**B (SE)**	***P* value**
Intercept	*3.46 (0.13)*	*<.001*	*3.46 (0.13)*	*<.001*	*3.53 (0.14)*	*<.001*
Time	0.54 (0.30)	.08	0.54 (0.30)	.08	0.66 (0.30)	.03
Condition	0.07 (0.19)	.72	-0.07 (0.19)	.72	0.14 (0.20)	.48
Timexcondition	0.12 (0.43)	.78	0.11 (0.43)	.80	0.01 (0.42)	.98

Note. Italicized values are statistically significant (P<.002)

Table S5 drug use (DAST-10)

**Fixed Effects**	**Comparison**					
	Human guided versus care as usual		Computer guided versus care as usual		Human guided versus computer guided	
**Parameter**	**B (SE)**	***P* value**	**B (SE)**	***P* value**	**B (SE)**	***P* value**
Intercept	*0.68 (0.07)*	*<.001*	*0.68 (0.07)*	*<.001*	*0.61 (0.07)*	*<.001*
Time	*0.74 (0.18)*	*<.001*	*0.74 (0.18)*	*<.001*	*0.72 (0.18)*	*<.001*
Condition	-0.07 (0.10)	.49	0.00 (0.10)	.99	-0.07 (0.10)	.49
Timexcondition	-0.02 (0.24)	.92	-0.16 (0.25)	.53	0.14 (0.23)	.55

Note. Italicized values are statistically significant (P<.002)

Table S6 subjective health (EQ-5D VAS)

**Fixed Effects**	**Comparison**					
	Human guided versus care as usual		Computer guided versus care as usual		Human guided versus computer guided	
**Parameter**	**B (SE)**	***P* value**	**B (SE)**	***P* value**	**B (SE)**	***P* value**
Intercept	*67.16 (0.85)*	*<.001*	*67.16 (0.85)*	*<.001*	*68.07 (0.86)*	*<.001*
Time	3.19 (3.05)	.30	3.19 (3.05)	.30	5.86 (2.96)	.05
Condition	0.91 (1.26)	.47	0.69 (1.27)	.59	0.22 (1.20)	.86
Timexcondition	2.67 (4.22)	.53	3.12 (4.42)	.48	-0.45 (4.36)	.92

Note. Italicized values are statistically significant (P<.002)
