# Supplementary material for: Web-Based, Human-Guided, or Computer-Guided Transdiagnostic Cognitive Behavioral Therapy in University Students With Anxiety and Depression: Randomized Controlled Trial
Source: JMIR Ment Health. 2024 Jun 19;11:e50503. doi: 10.2196/50503 (PMC11222767; doi:10.2196/50503)
Supplement: Multimedia Appendix 4 [file mental_v11i1e50503_app4.pdf]

Tables S1-S4 – Sensitivity analyses

Table S1 Long-term (6 months) results for depression of linear mixed models for pairwise comparisons of conditions for those with “sufficient eHealth”

**Fixed Effects**	**Comparison**					
	Human guided versus care as usual		Computer guided versus care as usual		Human guided versus computer guided	
**Parameter**	**B (SE)**	***P* value**	**B (SE)**	***P* value**	**B (SE)**	***P* value**
Intercept	*8.21 (0.29)*	*<.001*	*8.21 (0.29)*	*<.001*	*7.75 (0.33)*	*<.001*
Time	*4.53 (0.97)*	*<.001*	*4.53 (0.97)*	*<.001*	*7.63 (1.03)*	*<.001*
Condition	-0.47 (0.44)	.29	-0.27 (0.46)	.55	-0.19 (0.48)	.69
Timexcondition	3.10 (1.40)	.03	3.06 (1.46)	.04	0.04 (1.51)	.98

Note. Italicized values are statistically significant (P<.002)

Table S2 Long-term (6 months) results for anxiety of linear mixed models for pairwise comparisons of conditions for those with “sufficient eHealth”

**Fixed Effects**	**Comparison**					
	Human guided versus care as usual		Computer guided versus care as usual		Human guided versus computer guided	
**Parameter**	**B (SE)**	***P* value**	**B (SE)**	***P* value**	**B (SE)**	***P* value**
Intercept	*7.72 (0.26)*	*<.001*	*7.72 (0.26)*	*<.001*	*6.68 (0.29)*	*<.001*
Time	*3.41 (0.82)*	*<.001*	*3.41 (0.82)*	*<.001*	*6.23 (0.95)*	*<.001*
Condition	-1.04 (0.38)	<.01	-0.73 (0.42)	.09	-0.31 (0.44)	.48
Timexcondition	2.82 (1.19)	.02	2.59 (1.34)	.05	0.23 (1.41)	.87

Note. Italicized values are statistically significant (P<.002)

T**able S3** Long-term (6 months) results for depression of linear mixed models for pairwise comparisons of conditions for the unimputed dataset

**Fixed Effects**	**Comparison**					
	Human guided versus care as usual		Computer guided versus care as usual		Human guided versus computer guided	
**Parameter**	**B (SE)**	***P* value**	**B (SE)**	***P* value**	**B (SE)**	***P* value**
Intercept	*8.21 (0.22)*	*<.001*	*8.21 (0.22)*	*<.001*	*7.71 (0.22)*	*<.001*
Time	*2.88 (0.58)*	*<.001*	*2.88 (0.58)*	*<.001*	*6.23 (0.58)*	*<.001*
Condition	-0.49 (0.31)	.11	-0.06 (0.31)	.85	-0.43 (0.31)	.16
Timexcondition	*3.35 (0.82)*	*<.001*	*2.69 (0.82)*	*.<.01*	0.67 (0.82)	.42

Note. Italicized values are statistically significant (P<.002)

Table S4 Long-term (6 months) results for anxiety of linear mixed models for pairwise comparisons of conditions for the unimputed dataset

**Fixed Effects**	**Comparison**					
	Human guided versus care as usual		Computer guided versus care as usual		Human guided versus computer guided	
**Parameter**	**B (SE)**	***P* value**	**B (SE)**	***P* value**	**B (SE)**	***P* value**
Intercept	*7.78 (0.20)*	*<.001*	*7.78 (0.20)*	*<.001*	*6.67 (0.20)*	*<.001*
Time	*1.71 (0.51)*	*<.001*	*1.71 (0.51)*	*<.001*	*5.25 (0.51)*	*<.001*
Condition	*-1.11 (0.28)*	*.<.001*	-0.56 (0.28)	.50	-0.55 (0.28)	.53
Timexcondition	*3.54 (0.72)*	*.<.001*	*2.69 (0.72)*	*.<.001*	0.84 (0.72)	.24

Note. Italicized values are statistically significant (P<.002)
